# Cytologic Categorization with Risk Stratification of Endoscopic Ultrasound-Guided Fine Needle Aspiration from Pancreatic Lesions Based on Guidelines of the Papanicolaou Society of Cytopathology: 12-Year Tertiary Care Experience

**DOI:** 10.15190/d.2021.13

**Published:** 2021-08-21

**Authors:** Nilay Nishith, Ram Nawal Rao, Praveer Rai

**Affiliations:** ^1^Department of Pathology, Sanjay Gandhi Postgraduate Institute of Medical Sciences, Lucknow, UP, India; ^2^Department of Gastroenterology, Sanjay Gandhi Postgraduate Institute of Medical Sciences, Lucknow, UP, India

**Keywords:** EUS-FNA, pancreas, Papanicolaou Society of Cytopathology, risk of malignancy, risk stratification.

## Abstract

BACKGROUND AND AIMS: Pancreatic malignancy is an important cause of cancer mortality worldwide. Endoscopic ultrasound-guided fine-needle aspiration (EUS-FNA) plays a crucial role in the pre-operative diagnosis of pancreatic lesions. In this study, we have analyzed the cytological spectrum of pancreatic lesions in the Indian population over 12 years, categorized them according to the Papanicolaou Society of Cytopathology System for Reporting Pancreaticobiliary Cytology (PSCPC), and assessed the risk of malignancy (ROM) for each of the categories. METHODS: A computerized data search from January 2008 to December 2019 revealed 581 pancreatic EUS-FNA samples, among which surgical follow-up was available for 73 cases. All cytological specimens were reviewed and prospectively classified into one of the six diagnostic categories proposed by the PSCPC. Subsequently, a cytohistological correlation was performed and the ROM was calculated for each category. RESULTS: The cytologic diagnoses included 50 nondiagnostic (category I), 175 negative for malignancy (category II), 19 atypical (category III), 27 neoplastic:benign (category IVA), 30 neoplastic:other (category IVB), 26 suspicious (category V), and 254 malignant (category VI) cases. ROM for non-diagnostic aspirates, nonneoplastic benign specimens, atypical cases, neoplastic:benign, neoplastic:other, suspicious for malignancy, and the malignant category was 16.7%, 7.1%, 33.3%, 0.0%, 20.0%, 100%, and 78.6%, respectively. CONCLUSION: We document an increased risk of malignancy from category I to category VI of the PSCPC. The malignancy risk for category VI (malignant) was statistically significant in our study but was lower in comparison to the values reported by other authors. Nonetheless, such an approach would establish transparent communication between the pathologist and the clinician, as well as aid the clinician in decision making, particularly in intermediate categories.

## INTRODUCTION

Pancreatic cancer is recognized as the fourteenth most common type of malignancy, and the seventh leading cause of cancer mortality worldwide. Although the 5-year survival rate has minimally increased from 6 to 9% between 2014 to 2018, its prognosis remains dismal^1^. Therefore, a prompt and precise diagnosis of pancreatic malignancy is crucial to improve clinical outcomes. In this regard, multi-detector computed tomography (MDCT) is the best-validated imaging tool for investigating a suspected case of pancreatic cancer^2^. Additionally, endoscopic ultrasound-guided fine-needle aspiration (EUS-FNA) has high sensitivity and specificity for assessing the nature of pancreatic lesions. It is a safe, minimally invasive, and well-tolerated procedure that can rarely be complicated by needle track seeding^3^.

In 2014, Pitman et al. proposed the Papanicolaou Society of Cytopathology System for Reporting Pancreaticobiliary Cytology (PSCPC). It includes standardized terminology and a nomenclature scheme for pancreaticobiliary cytology. This system has been widely accepted as it unifies reporting terminology for transparent communication between clinicians and pathologists, reduces inter and intra-observer variability, provides maximum flexibility for patient management, and aids in risk stratification^4,5^. However, its applicability has been limited in the Indian sub-continent. Therefore, this study was undertaken to analyze the cytological spectrum of pancreatic lesions in the Indian population over a 12-year period, categorize them according to the PSCPC, establish cyto-histological correlation and assess the risk of malignancy (ROM) for each of the categories.

## MATERIALS AND METHODS

This retrospective study includes all the EUS-FNA of pancreatic lesions performed at our institute from January 2008 to December 2019. A total of 581 cases were retrieved via an electronic data search in the Hospital Information System. Among these, surgical follow-up was available for 73 cases. All the patients provided informed consent before the procedure. Also, the patient’s information was gathered according to the prepared checklist that included age, gender, site of aspiration, and histopathological follow-up. Cytological specimens from other abdominal lesions such as peri-pancreatic mass and lymph nodes or bile duct mass lesions were excluded from the study. During the procedure, both air-dried and ethanol-fixed smears were prepared from the aspirated material in each case. May-Grunwald-Giemsa stain was applied on air-dried smears whereas alcohol-fixed smears were reserved for Hematoxylin and eosin (H&E) stain and Papanicolaou (Pap) stain. All cytology cases were reviewed by an experienced cytopathologist (who was blinded to the histologic diagnosis) and prospectively classified into one of the six diagnostic categories according to the PSCPC, namely, non-diagnostic (category I), negative for malignancy (category II), atypical (category III), neoplastic (category IV), suspicious of malignancy (category V), and malignant neoplasm (category VI). The neoplastic category was further sub-divided into neoplastic:benign (category IVA) and neoplastic:other (category IVB). The cases were assigned to these categories based on criteria laid down by Pitman et al^4,6^. Histopathology was considered the gold standard for arriving at a final diagnosis. Immunohistochemistry was applied for cases as and when required. Follow-up histopathological confirmation of pancreatic adenocarcinoma, high-grade neuroendocrine tumors, mucinous neoplasms with high-grade dysplasia, pancreatoblastoma, undifferentiated carcinoma, myxofibrosarcoma, and lymphoma were regarded as malignant outcomes. Any metastatic malignancy to the pancreas was excluded from statistical analysis. For the ease of statistical evaluation, all the histological biopsies/specimens reported as “no evidence of granulomatous pathology or malignancy” and “inadequate for evaluation” have been categorized as benign.

**Table 1:** Clinicopathological profile of EUS-guided FNA from pancreatic lesions EUS: Endoscopic ultrasound; FNA: Fine-needle aspiration.

Parameters	Categories	Number of cases (n=581)	Percentage (%)
Age (in years)	<20	05	0.9
	21-40	85	14.6
	41-60	253	43.5
	61-80	218	37.5
	81-100	20	3.4
			
Gender	Males	366	63
	Females	215	37
			
Site	Head of pancreas	262	45.1
	Body of pancreas	24	4.1
	Tail of pancreas	15	2.6
	Uncinate process	8	1.4
	Pancreatic mass (site not specified)	272	46.8

Following cyto-histological assessment, the cytology cases were further sorted as true positives (diagnosed as malignant or suspicious of malignancy on both cytology and histopathology), true negatives (diagnosed with the absence of malignancy on both cytology and histopathology), false positives (diagnosed incorrectly as malignant or suspicious of malignancy on cytology) and false negatives (failure of diagnosis of malignancy on cytology). Thereafter, sensitivity, specificity, positive predictive value (PPV), and negative predictive value (NPV) were calculated. Furthermore, ROM and overall risk of malignancy (OROM) were also calculated for each of the diagnostic categories of the PSCPC. Absolute ROM is the ratio of cytology cases with malignant histopathology to the total number of cytology cases with follow-up histopathology for that category. Overall ROM is defined as the ratio of cytology cases with malignant histopathology to the total number of cytology cases with or without follow-up histopathology for that category. The statistical significance of the ROM was calculated using the Fisher exact test in comparison with non-neoplastic benign cases. A p-value of <0.05 was considered statistically significant.

## RESULTS

This study involves retrospective analysis of 581 EUS-guided pancreatic aspirates over 12 years in the Indian population. The age of the patients ranged from 14 to 99 years with a mean of 56.0 years and a standard deviation of +14.88 years. Majority (43.5%) of the patients belonged to the age group of 41-60 years. Among the 581 cases, 366 (63%) were males and 215 (37%) were females. The male to female ratio was 1.7:1. The site of aspiration was specified in 309 cytological specimens, amidst which the head of pancreas was the most common location for fine-needle aspiration in this study. The clinicopathological profile of pancreatic fine-needle aspirates has been summarized in Table 1.

**Figure 1:**
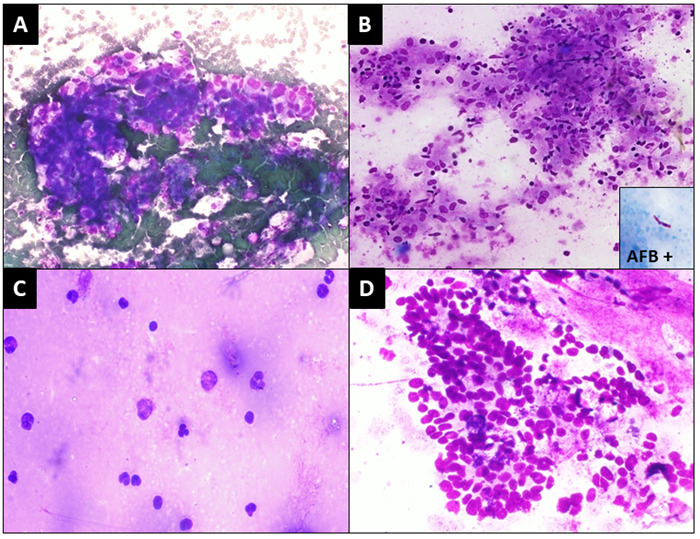
Cytological spectrum of pancreatic lesions **A. **Chronic pancreatitis: Benign pancreatic ductal epithelial cells with mild anisonucleosis embedded in hemorrhagic background (Giemsa; x400 magnification);** B. **Pancreatic Tuberculosis:Clusters of epithelioid histiocytes admixed with lymphocytes and necrosis (Giemsa; x400 magnification), inset: positive for acid-fast bacilli (Ziehl-Neelsen stain); **C. **Mucinous cystic neoplasm: Occasional benign epithelial cells on a mucinous background (Giemsa; x400 magnification); **D. **Suspicious for malignancy: Cluster of atypical epithelial cells with pleomorphic hyperchromatic nuclei (Giemsa; x400 magnification)

**Figure 2:**
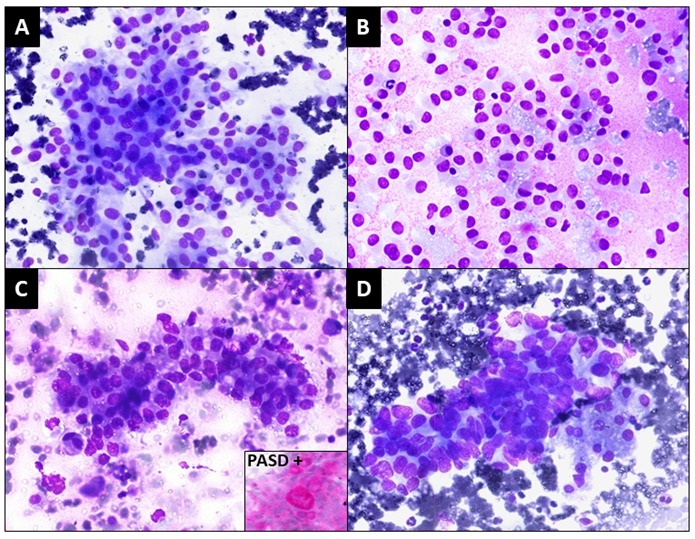
Cytological spectrum of pancreatic lesions **A.** Neuroendocrine tumour: Monomorphic tumour cells with stippled nuclear chromatin and scant cytoplasm (Giemsa; x400 magnification); **B.** Solid pseudo-papillary neoplasm: Dispersed population of round to oval cells with mildly pleomorphic hyperchromatic nuclei and nuclear grooving (Giemsa; x400 magnification); **C. **Well-differentiated adenocarcinoma: Acinar arrangement of tumour cells with nuclear pleomorphism (Giemsa; x400 magnification), inset: intracytoplasmic mucin globule (Periodic-acid Schiff stain after diastase predigestion); **D. **Metastatic adenocarcinoma (Giemsa; x400 magnification).

**Figure 3:**
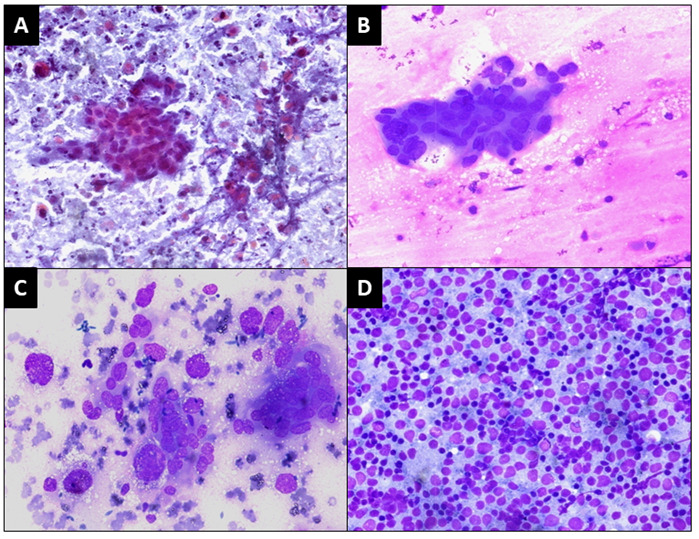
Cytological spectrum of pancreatic lesions **A.** Adenosquamous carcinoma: Three-dimensional cluster of tumour cells with interspersed dyskeratotic cells on a necrotic background (Pap; x400 magnification). **B.** Mucinous cystadenocarcinoma: Cluster of tumour cells on abundant extracellular pool of mucin (Giemsa; x400 magnification). **C. **Undifferentiated carcinoma: Loosely cohesive tumour cells with marked nuclear pleomorphism and multinucleation (Giemsa; x400 magnification). **D. **Non-Hodgkin lymphoma: Small to medium-sized atypical lymphoid cells with lymphoglandular bodies (Giemsa; x400 magnification).

On the categorization of all pancreatic EUS-FNA cases according to the PSCPC, the rate of non-diagnostic aspirations was 8.6% (50/581; category I). Negative for malignancy/category II accounted for 30.1% (175/581) with the most frequent being a negative report with a descriptive diagnosis (145/175) followed by pancreatitis (18/175) and pancreatic tuberculosis (08/175) (Figure 1, panels A and B). Category III/Atypical had 3.2% (19/581) cases. Both the sub-divisions of category IV had an almost equal number of cases. Category IVA (neoplastic:benign) comprised 4.6% (27/581) cases, out of which benign cystic neoplasm (23/27) was the most frequent diagnosis rendered and category IVB (neoplastic:other) included 30 cases (5.2%), which primarily constituted of neuroendocrine tumor (12/30) (Figure 2, panel A) followed by mucinous cystic neoplasm (07/30) (Figure 1, panel C) and solid pseudo-papillary neoplasm (04/30) (Figure 2, panel B). Category V/suspicious of malignancy (Figure 1, panel D) was allocated to 26 cases while the maximum cases (254/581; 43.7%) fell under category VI/malignant neoplasm. The most common malignant lesion was adenocarcinoma (230/254) (Figure 2, panel C) followed by adenosquamous carcinoma (06/254), mucinous cystadenocarcinoma (04/254), and poorly/undifferentiated carcinoma (2/254) (Figure 3, panels A, B and C). Rarely, we had encountered non-Hodgkin lymphoma (01/254) that comprised of small to medium-sized atypical lymphoid cells (Figure 3, panel D). Six cytological specimens diagnosed with non-pancreatic neoplasms (secondary malignancy) were excluded from statistical analysis; these cases included two lymphomas and one each of metastatic melanoma, metastatic neuroendocrine carcinoma, metastatic adenocarcinoma (Figure 2, panel D), and metastasis from an unknown primary. The categorization of all cases according to the PSCPC has been tabulated in Table 2.

Surgical follow-up was available in 73 (12.6%) cases among the 581 aspirates. However, the cyto-histopathological correlation was studied for 71 cases (excluding 2 histopathological cases of metastasis to the pancreas). As mentioned earlier, histopathology was considered the gold standard for arriving at a final diagnosis. In category I (Non-diagnostic), the discordance was observed in one of the six (16.7%) cases, wherein the cytology was reported as non-diagnostic, in contrast, the follow-up histopathological specimen was diagnosed as primary non-Hodgkin lymphoma (NHL). Again, two false-negative cases (02/28; 7.1%) were encountered in category II (Negative for malignancy). Both the cases were rendered a descriptive diagnosis on cytology; however, their histological examination revealed pancreatic ductal adenocarcinoma. The atypical/category III also showed one of three cases (33.3%), to be discordant with histology. There was no disagreement in category IVA (neoplastic: benign) and category V (suspicious for malignancy). While two cases (02/10; 20%) in category IVB (neoplastic:other) illustrated cyto-histological disparity; one being histologically diagnosed as myxofibrosarcoma and the other as ductal adenocarcinoma. The malignant category had three false-positive cases (03/14; 21.4%), which were cytologically labeled as positive for malignancy (suggestive of adenocarcinoma), though the excised specimens showed no evidence of malignancy and were validated as chronic pancreatitis. The details of these cases with cyto-histological correlation have been depicted in Table 2. Thus, the false-positive rate and false-negative rate noted in our study was 4.2% and 8.5%, respectively.

**Table 2:** Categorization of the EUS-guided pancreatic FNA according to PSCPC with histopathological correlation EUS: Endoscopic ultrasound; FNA: Fine-needle aspiration; IPMN: Intraductal papillary mucinous neoplasm; NET: Neuroendocrine tumor; NHL: Non- Hodgkin lymphoma; PSCPC: Papanicolaou Society of Cytopathology System for Reporting Pancreaticobiliary Cytology; WHO: World Health Organization.

Category according to PSCPC	Number of cases in each category (%)	Cytological diagnosis	Cytology (n=581) / Histopathol. (n=73) cases	Histopathological diagnosis
I. Non-diagnostic	50 (8.6%)	Non-diagnostic	50 / 06	Chronic pancreatitis (n=2), Lymphoplasmacytic sclerosing pancreatitis (n=1), Non-Hodgkin lymphoma (n=1), No evidence of granulomatous pathology or malignancy (n=2)
				
II. Negative for malignancy	175 (30.1%)	Pancreatitis (acute/chronic/auto-immune)	18 / 04	Chronic pancreatitis (n=3), Mucinous cystadenoma (n=1)
		Non-specific granulomatous inflammation /Tuberculosis	08 / -	-
		Pancreatic pseudocyst	03 / -	-
		Microfilariasis	01 / -	-
		Descriptive diagnosis	145 / 24	Benign epithelial cyst (n=1), Chronic pancreatitis (n=10), Granulomatous inflammation (n=1), Pancreatic pseudocyst (n=2), Benign mesenchymal tumor (n=1), Ganglioneuroma (n=1), Adenocarcinoma (n=2), NET (WHO grade 1) (n=1), No evidence of granulomatous pathology or malignancy (n=4), Inadequate for evaluation (n=1),
				
III. Atypical	19 (3.2%)	Atypical cells present	19 / 03	No evidence of granulomatous pathology or malignancy (n=2), Adenocarcinoma (n=1)
				
IV A. Neoplastic: Benign	27 (4.6%)	Benign cystic neoplasm	23 / 09	Mucinous cystadenoma (n=2), Borderline mucinous cystadenoma (n=2), Serous cystadenoma (n=2), IPMN (n=1), Chronic atrophic pancreatitis (n=1), Inadequate for evaluation (n=1)
		Benign, NOS	04 / -	-
				
IV B. Neoplastic:other	30 (5.2%)	Solid pseudopapillary neoplasm	04 / 02	Solid pseudopapillary neoplasm (n=2)
		Neuroendocrine tumour (NET)	12 / 04	NET (WHO Grade 2) (n=4)
		Mucinous cystic neoplasm	07 / 02	Myxofibrosarcoma (n=1), Adenocarcinoma (n=1)
		Intraductal papillary mucinous neoplasm (IPMN)	01 / 01	No evidence of granulomatous pathology or malignancy (n=1)
		Mesenchymal neoplasm	06 / 01	No evidence of granulomatous pathology or malignancy (n=1)
V. Suspicious for malignancy	26 (4.5%)	Suspicious for malignancy	26 / 01	Adenocarcinoma (n=1)
				
VI. Malignant	254 (43.7%) ­	Adenocarcinoma	230 / 12	Adenocarcinoma (n=8), Anaplastic carcinoma (n=1), Chronic pancreatitis (n=3)
		Adenosquamous carcinoma	06 / -	-
		Mucinous cystadenocarcinoma	04 / -	-
		Poorly /Undifferentiated carcinoma	02 / -	-
		Small cell neuroendocrine carcinoma	01 / -	-
		Pancreatoblastoma	01 / -	-
		Burkitt Lymphoma/Non-Hodgkin lymphoma	02 / 02	Burkitt lymphoma (n=1), T-cell NHL (n=1)
		Lympho-proliferative disorder	02 / -	-
		Metastasis to pancreas	06 / 02	Colonic mucinous adenocarcinoma with jejunal and pancreatic infiltration (n=1), Inadequate for evaluation (n=1)

Table 3 demonstrates the sensitivity, specificity, PPV, and NPV when considering a malignant (category VI) cytologic diagnosis as a positive test result; when considering malignant (category VI) and suspicious (category V) cytologic diagnoses as positive test results; when considering malignant (category VI), suspicious (category V) and neoplastic:other (category IVB) cytologic diagnoses as positive test results; and when considering malignant (category VI), suspicious (category V), neoplastic:other (category IVB) and atypical (category III) cytologic diagnoses as positive test results.

**Table 3:** Sensitivity, Specificity, PPV, and NPV of the PSCPC diagnostic categories in the present study PPV: Positive predictive value; NPV: Negative predictive value; PSCPC: Papanicolaou Society of Cytopathology System for Reporting Pancreaticobiliary Cytology.

Diagnostic category(ies) considered as a positive test result	Sensitivity (%)	Specificity (%)	PPV (%)	NPV (%)
Malignant (category VI)	73.3	98.7	78.6	98.3
Suspicious (category V) and malignant (category VI)	75.0	98.9	80.0	98.6
Neoplastic:other (category IVB) plus suspicious (category V) plus malignant (category VI)	83.3	98.1	80.0	98.5
Atypical (category III) plus neoplastic:other (category IVB) plus suspicious (category V) plus malignant (category VI)	84.6	97.8	81.5	98.4

The absolute ROM and OROM with p-value (relative to benign ROM) for each of the diagnostic categories of the PSCPC have been tabulated in Table 4. The risk of malignancy for category VI (malignant) was found to be statistically significant (p<0.001).

**Table 4:** Absolute and overall risks of malignancy of the PSCPC diagnostic categories in the present study *Statistically significant (p-value <0.05) PSCPC: Papanicolaou Society of Cytopathology System for Reporting Pancreaticobiliary Cytology; ROM: Risk of malignancy.

PSCPC category	Absolute ROM, %	Overall ROM, %	p-value (relative to benign ROM)
I	16.7	2.0	0.45
II	7.1	1.1	-
III	33.3	5.3	0.27
IVA	0.0	0.0	1.0
IVB	20.0	6.6	0.56
V	100.0	38.5	0.10
VI	78.6	4.5	<0.001*

## DISCUSSION

EUS-FNA has excellent accuracy in detecting malignant pancreatic neoplasms. Various meta-analyses have documented a pooled sensitivity for the diagnosis of malignancy on cytology ranging from 85.0% to 94.0%, and a pooled specificity ranging from 95.0% to 99.3%, respectively; particularly for solid lesions^7-11^. On the contrary, the meta-analysis authored by Wang et al^12^ exclusively analyzed EUS-FNA of pancreatic cystic lesions and demonstrated a drastic fall in pooled specificity i.e. 54%, while the pooled sensitivity was 94%; which was similar to the ranges indicated for solid pancreatic lesions. The results of the present study encompass data over a period of 12 years and have included all the spectrum of pancreatic lesions may they be solid, solid-cystic, and purely cystic lesions. In this study cohort, the specificity range falls in accordance with the previous studies. However, the sensitivity was slightly lower as compared to formerly reported values. The specificity was observed to be the highest at 98.9% when the suspicious (category V) and malignant (category VI) cytologic diagnoses were included in the positive test results, while sensitivity was the highest at 84.6% considering atypical (category III), neoplastic:other (category IVB), suspicious (category V) and malignant (category VI) as positive test results (Table 3). These results were concordant with Hoda et al^13^, who reported a sensitivity ranging from 66.2% to 99.2% and a specificity ranging from 61.7% to 100%.

**Table 5:** Comparison of absolute ROMs (%) of the PSCPC diagnostic categories in various reported studies PSCPC: Papanicolaou Society of Cytopathology System for Reporting Pancreaticobiliary Cytology; ROM: Risk of malignancy; NA: Not available.

PSCPC category	Current study	Sung et al 2020^14^	Hoda et al 2019^13^	Chen et al 2017^15^	Wright et al 2017^16^	Layfield et al 2014^16^
I	16.7	25.0	7.7	57.1	33.3	21.4
II	7.1	17.4	1.0	18.1	8.3	12.6
III	33.3	41.8	28.0	69.2	100.0	73.9
IVA	0.0	0.0	0.0	NA	66.7	NA
IVB	20.0	34.3	30.3	20.0	100.0	14.2
V	100.0	95.5	100.0	87.5	100.0	81.8
VI	78.6	99.6	100.0	100.0	100.0	97.2

To the best of the authors’ knowledge, this is the first study from the Indian sub-continent assessing the risk of malignancy associated with the diagnostic categories defined by the PSCPC. Our study reported an increasing malignancy risk from category I (Non-diagnostic) to category VI (malignant). The highest absolute risk of malignancy is noted in category V (suspicious) and category VI (malignant). This observation appears to mirror the findings of Sung et al^14^, Chen et al^15^, and Layfield et al^17^ (Table 5). But the current study reports a greater ROM for the suspicious category in comparison to the malignant category. While the other investigators (Sung et al, Chen et al, and Layfield et al) revealed the ROM for both category V (suspicious) and category VI (malignant) to be the same or a higher ROM for category VI (malignant). The risk of malignancy becomes primarily important for indeterminate categories, as they aid in the clinical management of the patient. Wright et al^16^ also described their experience using the PSCPC for EUS-FNA of both solid and cystic pancreatic lesions. Their ROM for the respective diagnostic categories has also been included in Table 5. They reported a 100% ROM for atypical (category III), as well as neoplastic:other (category IVB); and a 66.7% ROM for neoplastic:benign category. This was the cardinal dissimilarity with the present study, wherein the ROM for indeterminate categories was lower and ranged between 0.0-33.3%. The over-estimation of ROMs in the indeterminate categories by Wright et al^16^ may be attributed to the difference in the definition of the malignant outcome, as they had included all neoplasms with malignant potential, including low-grade mucinous neoplasms and neuroendocrine tumors as malignant. Chen et al^15^calculated ROM based on re-categorization of prior EUS-FNA cases according to the PSCPC, whereas Layfield^17^ analyzed a total of 317 EUS-FNAs with either surgical follow-up or had a clinical follow-up of >3years to determine the outcome. In both the studies, the neoplastic categories (category IV, neoplastic:benign, and category IV neoplastic:other) were combined into one category (i.e. category IV: neoplastic) for the calculation of ROM. The reported ROM from the studies by Chen et al^15^ and Layfield et al^17^ for the neoplastic category was 20% and 14.2%, respectively. On the contrary, Hoda et al^13^ and Sung et al^14^ stratified category IVB (neoplastic:other) into low-grade dysplasia (LGD) and high-grade dysplasia (HGD). The ROM for category IVB was 30.3% and 34.3% as reported by Hoda et al^13^ and Sung et al^14^, respectively. Surprisingly, both the studies demonstrated a three-fold increase in ROM in the category IVB with high-grade dysplasia. Therefore, the distinction between the two sub-classes of neoplastic:other (category IVB) is important as both the categories project differences in risk of malignancy and prognostic implications. This has also been substantiated by Smith et al^18^ and Hoda et al^19^, who assessed the ROM in mucinous cystic neoplasms of the pancreas and concluded that mucinous cysts with HGD confer a much greater ROM and therefore, warrants surgical resection. Additionally, Majumder et al^20^ and Rezaee et al^21^ established an increased risk of concurrent and subsequent ductal adenocarcinoma with mucin-producing cystic neoplasm of the pancreas; predominantly intraductal papillary mucinous neoplasm (IPMN). Therefore, we amongst others strongly recommend sub-classification of neoplastic:other (category IVB) into neoplastic:other with LGD and neoplastic:other with HGD.

**Figure 4:**
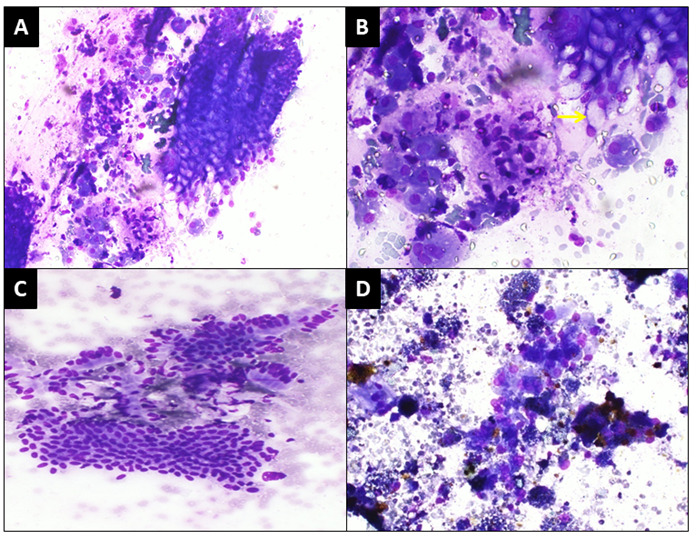
Non-neoplastic elements in pancreatic cytology **A. **Honeycomb pattern of normal duodenal mucosa with adjoining benign pancreatic acini (Giemsa; x200 magnification). **B. **Goblet cell (arrow)(Giemsa; x400 magnification). **C.** Picket-fence arrangement with basally located nuclei of normal duodenal epithelial cells (Giemsa; x400 magnification). **D.** Clusters of benign pancreatic acini admixed with hemosiderin-laden macrophages on a hemorrhagic background (Giemsa; x400 magnification).

We report a false-negative rate of 8.5% for EUS-FNA of pancreatic lesions, which was comparable to the studies published in the recent past and ranged from 1% to 25%^13,16,19,22-24^. Most of the false-negative cases in our study resulted from sampling error. Woolf et al^24^ evaluated a total of 733 EUS-FNA of pancreatic solid and cystic lesions and established that sampling errors followed by interpretive errors were the most common causes for false-negative diagnoses. Besides these, the presence of gastric and duodenal epithelium with goblet cells (which are usually arranged in honeycomb/picket-fence pattern) (Figure 4, panels A, B and C), hemosiderin-laden macrophages (Figure 4, panel D), and gastrointestinal tract mucin may be mistaken for mucinous cystic neoplasm^25^.On the other hand, the false-positive rate was 4.2% in our study, which was again in agreement with those reported by other investigators^13,16,19,22,23^. A large institutional study conducted by Gleeson et al^26^highlighted that false-positive diagnoses in EUS-FNA were often encountered in a setting of Barrett’s esophagus with dysplasia, early malignancy, chronic or autoimmune pancreatitis, and reactive gastropathy. Likewise, for all the three false-positive cases in our study, the ensuing histologic specimens were reported as chronic pancreatitis. A review article authored by Michelle Reid^25^ states that chronic pancreatitis by virtue of its cytomorphology can often be confused with ductal adenocarcinoma. In this regard, the final cytology report should be rendered following a meticulous clinico-radiological and cytological correlation.

Apart from being a single-center retrospective study, one of the major drawbacks of our series is the paucity of the follow-up histopathological specimens. A possible explanation for such a limitation is that majority of our patients may not undergo surgery, either because, they have lesions that can be managed clinically without any surgical intervention or they have large unresectable masses with a poor prognosis at the time of initial diagnosis. Besides, as our institution is a tertiary care and referral center, hence, some patients choose to undergo primary diagnostic work-up here but end up opting for surgery at a specialized oncology institute. About the malignancy risk, we have reported an increase from category I (non-diagnostic) to category VI (malignant). The ROM for the malignant category was statistically significant but was lower in comparison to the values reported by other authors. The malignancy risk for the rest of the diagnostic categories of the PSCPC was not statistically significant. Therefore, we advocate large-scale multi-institutional Indian studies to confirm the risk stratification demonstrated in the current study.

## CONCLUSION

The diagnostic categories laid down by the PSCPC for categorization of EUS-FNA cytology of pancreatic lesions has been widely accepted as it provides flexibility for patient management and aids in risk stratification. In this study, we have reviewed and prospectively classified all the 581 cases according to the PSCPC and demonstrated an increasing malignancy risk from category I to category VI. Such a risk stratification strategy would unify the reporting terminology across the globe and assist the clinician in decision making, particularly in intermediate categories. Also, we encourage the sub-classification of neoplastic:other category into neoplastic:other with LGD and neoplastic:other with HGD as each of them confer different prognostic significance. However, our study is limited by the paucity of surgical follow-up specimens. Therefore, we advocate large-scale multi-institutional Indian studies to confirm the risk stratification demonstrated in the current study.
